# Supplementary material for: Heterogeneity of malaria transmission in urban settings in Ethiopia: A seroprevalence and risk factor analysis
Source: PLoS One. 2026 Feb 5;21(2):e0328118. doi: 10.1371/journal.pone.0328118 (PMC12875449; doi:10.1371/journal.pone.0328118)
Supplement: S1 Table — (DOCX) [file pone.0328118.s003.docx]

**Supplementary Tables and Definition of variables**

**Supplemental Table 1. List of coupled malaria antigens**

| **Gene Id** | **Antigen name** | **Batch** | **Stock concentration µg/ml** | **EC50 (ug/6.25e6 beads)** | **Coupling volume (µl)** | **Volume Ag (µl)** | **PBS volume (µl)** | **Supplier** | **Description** |
| --- | --- | --- | --- | --- | --- | --- | --- | --- | --- |
|  | Pv DBP RII | Pasteur 22 | 1200 | 1.114 | 750 | 1.39 | 1498.6 | LSHTM | Region II of *Plasmodium vivax* Duffy binding protein |
|  | Pv EBP | Pasteur  160415 | 1000 | 0.38114 | 750 | 0.57 | 1499.4 | LSHTM | Pv erythrocyte binding protein genes |
|  | PvRBP2b | Pasteur 13/01/16 | 1100 | 2.06512 | 750 | 2.82 | 1497.2 | LSHTM | Pv reticulocyte binding protein 2b |
|  | PvAMA1 | 270815 | 778 | 5.8 | 750 | 11.18 | 1488.8 | LSHTM | Pv apical membrane antigen |
|  | PvMSP1 19 | KT 160518 | 13650 | 190 | 750 | 20.88 | 1479.1 | LSHTM | Pv merozoite surface protein |
| PF3D7_0930300 | PfMSP1 19 | Dialysed 181017 | 2370 | 51.5 | 750 | 32.59 | 1467.1 | LSHTM | *Plasmodium falciparum* merozoite surface protein |
|  | PfAMA1 | KT 160518 | 2010 | 3.85 | 750 | 2.87 | 1497.1 | LSHTM | Pf apical membrane protein |
| PF3D7_1035300 | GLURP R2 | Thessein | 800 | 0.042 | 750 | 0.08 | 1499.9 | LSHTM | Glutamate rich protein R2 |
| PF3D7_0423700 | Etramp5.Ag1 | KT 120917 | 10360 | 32.5 | 750 | 4.71 | 1495.3 | LSHTM | Early transcribed membrane protein 5 |
| PF3D7_0501100.1 | HSP40.Ag1 | 200917 | 6270 | 91.5 | 750 | 21.89 | 1478.1 | LSHTM | Heat shock protein 40, type II |
|  | TT | 02/232 | 5000 | 35 | 750 | 10.5 | 1489.5 | LSHTM | Tetanus Toxoid |
|  | GST | 150917 | 22600 | 29 | 750 | 1.92 | 1498.1 | LSHTM | GTS express tag |
