# Supplementary material for: Heterogeneity of malaria transmission in urban settings in Ethiopia: A seroprevalence and risk factor analysis
Source: PLoS One. 2026 Feb 5;21(2):e0328118. doi: 10.1371/journal.pone.0328118 (PMC12875449; doi:10.1371/journal.pone.0328118)
Supplement: S2 Table — (DOCX) [file pone.0328118.s004.docx]

**Supplemental Table 2. Seroprevalence of malaria by species, antibody, and town (with 95%CI) for individuals less than 15 years of age.**

|  | | **Adama %(CI)** | **Awash Sebat Kilo %(CI)** | **Metehara %(CI)** |
| --- | --- | --- | --- | --- |
| **Pf Short Lived** | Etramp5.Ag1 | 3.2(1.6-6.3) | 22.4(17.8-27.9) | 13.3(9.6-18.0) |
|  | GEXP18 | 2.0(0.8-4.7) | 28.5(23.4-34.3) | 14.8(10.9-19.8) |
|  | HSP40.Ag1 | 0.4(0-2.8) | 18.3(14.0-23.4) | 9.4(6.4-13.6) |
| **Pf Long lived** | PfMSP119 | 2(0-4.7) | 45.6(39.7-51.7) | 28.5(23.3-34.4) |
|  | PfGLRUP2 | 4.4(2.5-7.8) | 46.8(40.8-52.8) | 30.1(24.8-36) |
|  | PfAMA1 | 0.8 (0.2-3.1) | 25.9(20.9-31.5) | 18.8(14.4-24.0) |
| **Pv Short lived** | MSP1-19 | 1.6(0.6-4.2) | 15.2(11.3-20.1) | 16.4(12.3-21.5) |
|  | PvEBP | 0 | 6.8(4.3-10.6) | 7.4(4.8-11.4) |
| **Pv Long lived** | PvAMA1 | 2.8(1.3-5.8) | 21.3(16.7-26.7) | 10.9(7.6-15.4) |
|  | PvDBP RII | 1.6(0.6-4.2) | 14.1(10.3-18.8) | 11.3(7.9-15.8) |
