# Supplementary material for: Heterogeneity of malaria transmission in urban settings in Ethiopia: A seroprevalence and risk factor analysis
Source: PLoS One. 2026 Feb 5;21(2):e0328118. doi: 10.1371/journal.pone.0328118 (PMC12875449; doi:10.1371/journal.pone.0328118)
Supplement: S1 File — (DOCX) [file pone.0328118.s005.docx]

**Definitions of variables**

Improved water source: dug well or piped water, or bottled water

Improved sanitation: flush or pour toilet; or Pit latrine

Improved cooking fuel: use of electricity and/or Kerosin

Improved floor material: made of cement, ceramic or polished wood

Improved roof material: made from metal, or wood or cement

Footnote for Supplemental Fig 1: A different scale was chosen for Adama to ensure data visibility.
