# Supplementary material for: Heterogeneity of malaria transmission in urban settings in Ethiopia: A seroprevalence and risk factor analysis
Source: PLoS One. 2026 Feb 5;21(2):e0328118. doi: 10.1371/journal.pone.0328118 (PMC12875449; doi:10.1371/journal.pone.0328118)
Supplement: Attachment — Submitted filename: My feedback for Manuscript Number PONE-D-25-32925.docx [file pone.0328118.s006.docx]

**My Feedback for PLOS One**

**MS title: Heterogeneity of Malaria in Urban Settings in Ethiopia: A Seroprevalence and Risk
Factor Study.**

**Corresponding Author: Hiwot Teka Namaga**, Addis Ababa University, Addis Ababa, ETHIOPIA

**My general reflection on the Work**

The authors have produced an interesting, a highly exhaustive, timely, and impactful study on emerging challenges related to urban malaria in Ethiopia. This work has the potential to significantly contribute to the ongoing malaria elimination efforts in the country.

**Section I: Minor Revision Sections**

1. **Comments on the title of the Manuscript (MS):**
A. However, I recommend changing the title to **'Heterogeneity of Urban Malaria in Ethiopia: A Seroprevalence and Risk Factor Analysis**”. Using the term "*Analysis*" instead of "Study" is academically precise. It's also understood that the manuscript is a study, and "**Analysis**" implies a focused investigation of the data.
B. Please note that seroprevalence should be written as one single, unhyphenated word
2. **Comments on the Abstract Section:**

In general, the abstract is well-written and scientifically sound. However, the following key details are missing or require clarification:

A. **Problem Statement (Line 20-21)**: The introductory sentences need to clearly establish the context and challenges of urban malaria in your study setting. While you introduced the topic sentence, you didn’t t explicitly state why the study was needed. **Please revise and add a sentence that concisely articulates (with only one sentence)**:
   - The specific problem or challenge associated with urban malaria transmission/control.
   - Why using a seroprevalence and risk factor approach is necessary to address that challenge.
B. **Objective statement (Line 21):** Please change the phrasing from simple present tense "**This study aims**..." to the past tense as "**This study aimed to**...”
C. **Method section (Line 22-24):** Please specify the sampling technique used to select the individuals (participants) from within the 277 selected households. It is crucial to clarify how individuals were chosen from each household.
D. **Line 25:** Write the phrase as quantitative polymerase-chain reaction (qPCR). You should then use the abbreviation qPCR consistently throughout the remainder of your MS.
E. **Result section:** Please provide total seroprevalence rates, as shown in the RDTs and PCR results, and then proceed to each town as described above.
   - **Line 26-27**: Your study found a low malaria prevalence (0.78% by RDT and 4% by qPCR). The study period (January 30 to March 14, 2020) corresponds to the dry season in Ethiopia (approximately February to March), a period typically characterized by low malaria transmission.
   - **Please connect the following points in your analysis**:
     - Wasn't low malaria prevalence expected?
     - What reference establishes this prevalence as "low"?
     - What were the status of your participants? Were they malaria patients?
   - **Line 30-31:** Please include the AOR and 95% CI for the significant associations of risk factors. This is one of the areas your audience want to read.
   - **Conclusion:** Your Abstract lacked *conclusion* based on your findings. The first sentence appears recommendation. *Please revise this section to first provide a concise, interpretive conclusion that directly summarizes the main implication(s) of your results*.
3. **Comments on the main MS:**
A. **Method section**: Study area is well written and excellent. You may connect my previous questions (low malaria transmission period in the country to the local contexts).
   - **Line 98**: **Figure 1: Map of the study sites (26):** Reference 26 is formatted incorrectly. Please use the standard reference style for websites and ensure it conforms to the journal's guidelines. Alternatively, you may source and cite the map from official national data.
   - **Additionally**, reference 25 is also incorrect. For example, the "**Ethiopian Statistical Agency**" should be updated to its current name, the "**Ethiopian Statistical Service**."
   - **Line111-116: Please use this subtitle as “Sampling Design, Sampling technique and Study Population”**:
     - **Line 112**: Please start with: **A community-based** cross-sectional survey was conducted between January 30….. Because you collected your data from the community (included individuals from households). People who are reading your work will pay attention. Make sure also you stated in a similar fashion in the abstract section.
     - **Sampling technique**: Please include whether you used a ***Systematic random sampling*** (SRS) in this section. It makes this section complete and readable.
     - **Line 113**: please replace “**parasitemia**” with “**infection”**.
     - **Sample size determination: Not clear.**  Please detail the methodology used to arrive at 277 households. Furthermore, explain the steps for selecting 912 individuals from those 277 households and three towns.
     - **Line 125-:** Please clearly include **inclusion** and **exclusion** criteria of your study participants. This will enhance clarity of your study.
   - **Line 138 (Section Molecular and Serological analyses),** please remove the word “conducted “.
   - **Line 163 -168** likely address sample size determination, which is a prerequisite planning step and ***not a part of the statistical analysis conducted on your collected data***. You should therefore cut this content from Lines 164–168, create a new subheading titled "**Sample size determination**," and paste this entire section between line numbers 124 and 125 to improve the flow and organization of your Methods section.
   - **The Statistical analysis section** should start from line 169**.**
   - **Line 170:** Please write out the full term for the abbreviation Finite Mixture Models (FMM) upon its first mention.
   - **Line 172:** as suggested above, do the same for MFI.
   - **Line 182-187**: please provide reference (citation) for both statistical models you used.
   - **Line 202 and 204**: Please provide valid references for the softwares you used. This s acknowledging their works.
B. **Results section:**
   - **Line 216:** Kindly replace the subheading "Household and Participant Characteristics" with the more precise phrase: "Household and Sociodemographic Characteristics of the Study Participants**.**
   - **In this result section,** it is standard practice to present the Sociodemographic Characteristics of the Study Participants before presenting the general Household Characteristics. Please reorder your content accordingly. Specifically, the table currently labeled Table 1 should be renumbered as Table 2, and the table currently labeled Table 2 should become Table 1. So, your paragraph one must Sociodemographic Characteristics of the Study Participants and etc.
   - **Line 232:** Please correct as: A total of 912 individuals were included in the surveyed households, of whom 51.2% (n=467) were male.
   - **Line 240:** rewrite the subheading as **Prevalence of malaria infection.**
   - **Line 244:** 2.3%P. vivax. Please press the pace bar between % and P. vivax as 2.3% P. *vivax.*
   - **Line 250:** Why the total number of individuals **included in your Serological testing was 767? Whynot 774? Please justify this?**
   - **Line 250:** Please include the seroprevalence of malaria as 19.4% (n=767) in your abstract section.
   - **Line 260:** Please correct the caption for Table 3. The current caption, "Fever and malaria prevalence," is too brief. Please follow standard academic format by including the essential context (e.g., location, time, or participant group). For example: *Table 3. Fever and prevalence of malaria in selected towns within Eastern Oromia Regional State, Ethiopia, January to March, 2020*.
C. **Discussion Section:**
   - It is well written; result interpretations and pertinent justifications are provided.
   - **Line 407-412: Studies have documented widespread**... The claim of "widespread documentation" is currently supported by only a single reference (68). Please strengthen this statement by adding **multiple relevant citations** besides number 68. At the same time, please go through this section and strengthen your synthesis ans justifications with more references (citations).
4. **Important questions**

**Line 419-422: Your recommendations: “**To address these challenges, malaria control programs must adopt more adaptive, data-driven strategies. This includes deploying next-generation tools such as dual-active ingredient nets, rotating insecticides for IRS, and enhancing community education to promote consistent ITN use.”

A. Is this the only recommendation from your study for the National malaria control programme of Ethiopia?
B. Don’t you think what you are recommending are already not in place?
C. Your findings show that RDT-based prevalence of malaria is only 0.78% (n=6) while that qPCR is 4% (n=31).
   1) The data show a significant discrepancy: RDT-based prevalence is very low at 0.78% (n=6), yet qPCR-based prevalence is five times higher at 4% (n=31). Given these findings, what does this mean for the operational goals of the current National Malaria Elimination Strategic Plan (2024/25-2026/27)? Specifically, how might this "hidden reservoir" of infection (detected only by qPCR) affect elimination targets and surveillance strategies relying primarily on RDTs?
   2) What is the likely scientific and/or biological explanation for the five-fold difference in prevalence between the two diagnostic tools?
   3) Based on the above analysis, what specific recommendations do you propose regarding diagnostic policy and surveillance strategies in the context of the National Malaria Elimination Plan? Ensure these points are explicitly integrated into the Discussion and Recommendation sections of your manuscript.
5. **General comments:**

**Referencing and Citation Style: P**lease conduct a thorough, final review of the entire References section and in-text citations to ensure absolute consistency. All necessary corrections should be implemented as detailed in the previous specific comments regarding style (e.g., journal name abbreviations, punctuation, and author formatting.

**Manuscript Page Numbering:** For ease of review and standard formatting, please ensure that all page numbers are consistently positioned at the bottom-center of every page.

My overall conclusion about the MS

Overall assessment: The manuscript is requiring **only minor revisions**. The authors have presented a sound study that requires only minor adjustments to clarity, formatting, and the discussion of key findings. And Answer to some questions.
