# Supplementary material for: Heterogeneity of malaria transmission in urban settings in Ethiopia: A seroprevalence and risk factor analysis
Source: PLoS One. 2026 Feb 5;21(2):e0328118. doi: 10.1371/journal.pone.0328118 (PMC12875449; doi:10.1371/journal.pone.0328118)
Supplement: Attachment — Submitted filename: Response to reviewers.docx [file pone.0328118.s009.docx]

**Response to Reviewer #1**

Section I: Minor Revision Sections

1. Comments on the title of the Manuscript (MS):

A. However, I recommend changing the title to 'Heterogeneity of Urban Malaria in Ethiopia: A Seroprevalence and Risk Factor Analysis”. Using the term "Analysis" instead of "Study" is academically precise. It's also understood that the manuscript is a study, and "Analysis" implies a focused investigation of the data.

B. Please note that seroprevalence should be written as one single, unhyphenated word

We accept the suggestion of replacing the word "Study" by "Analysis" and unhyphenating seroprevalence, the suggested changes are shown in the title (Line #1-3)

2.	Comments on the Abstract Section:

In general, the abstract is well-written and scientifically sound. However, the following key details are missing or require clarification:

A.	Problem Statement (Line 20-21): The introductory sentences need to clearly establish the context and challenges of urban malaria in your study setting. While you introduced the topic sentence, you didn’t t explicitly state why the study was needed. Please revise and add a sentence that concisely articulates (with only one sentence):

O The specific problem or challenge associated with urban malaria transmission/control.

O Why using a seroprevalence and risk factor approach is necessary to address that challenge.

We appreciate the comment and added clarifying sentences to reflect the problem statement and the importance of seroprevalence in assessing risk factors. The following statements are added in the revised manuscript “In the urban settings, malaria has become a concern due to rapid unplanned urbanization, poor drainage, and migration that create breeding sites for vectors such as *Anopheles stephensi* leading to complex transmission patterns that underestimate the malaria risk” (Line #23-25)

Risk factor analyses using serological indicators provides insight into patterns of malaria transmission and exposure, and can be used to identify drivers of transmission, and enable design of more tailored interventions.” (Line #27-30)

B.	Objective statement (Line 21): Please change the phrasing from simple present tense "This study aims..." to the past tense as "This study aimed to...”

Thank you for flagging the grammar correction, the manuscript is edited (Line #26).

C.	Method section (Line 22-24): Please specify the sampling technique used to select the individuals (participants) from within the 277 selected households. It is crucial to clarify how individuals were chosen from each household.

We used randomization for selecting the households. (Line #31) All participants age above 6 months who provided consent were included from the selected households. We have made edits in the abstract to clarify the statement. (Line #139)

D.	Line 25: Write the phrase as quantitative polymerase-chain reaction (qPCR). You should

then use the abbreviation qPCR consistently throughout the remainder of your MS.

We have accepted the suggestion and made the recommended changes. (Line #32)

E.	Result section: Please provide total seroprevalence rates, as shown in the RDTs and PCR results, and then proceed to each town as described above.

We have accepted the suggestion and added the overall seroprevalence rates in the revised manuscript. (Line #35-37)

–	Line 26-27: Your study found a low malaria prevalence (0.78% by RDT and 4% by qPCR). The study period (January 30 to March 14, 2020) corresponds to the dry season in Ethiopia (approximately February to March), a period typically characterized by low malaria transmission.

–	Please connect the following points in your analysis:

o	Wasn't low malaria prevalence expected?

Yes, we expected the low prevalence in these settings in the minor season and in urban settings.

o	What reference establishes this prevalence as "low"?

The detailed stratification information is included in the methods section of the manuscript. As per the WHO stratification low transmission is defined as area with Annual Parasite Incidence of less than100 per 1000 population (Line #120-124). (Reference: WHO. Malaria surveillance, monitoring & evaluation: a reference manual 2018. 105 p.). Ethiopia uses its own national stratification defined as below table (Reference: FMOH. Ethiopia Malaria Elimination Strategic Plan: 2021-2025. Addis Ababa, Ethiopia: The Federal Democratic Republic of Ethiopia Ministry of Health; 2020. p. 88.).

| **Malaria endemicity** | **National Classification** | **WHO Classification** |
| --- | --- | --- |
| High | API>=50 | API>450 |
| Moderate | API>=10&<50 | API 250-450 |
| Low | API>5 &<10 | API 100-250 |
| Very low | AP>0 &<=5 | API <100 |
| Free | API=0 |  |

o	What were the status of your participants? Were they malaria patients?

No, the study participants were residents within random sample of households and they were not malaria patients. We did community survey. (line # 131-138)

–	Line 30-31: Please include the AOR and 95% CI for the significant associations of risk factors. This is one of the areas your audience want to read.

We accepted the suggestion and made revision as it is suggested (Line #40-41).

–	Conclusion: Your Abstract lacked conclusion based on your findings. The first sentence appears recommendation. Please revise this section to first provide a concise, interpretive conclusion that directly summarizes the main implication(s) of your results.

We appreciate the comment and rephrased the first statement as follows *“This study identified the lowest seroprevalence level in Adama and higher levels in Awash Sebat Kilo highlighting the heterogeneous nature of malaria transmission in the urban settings”* . (Line #42-48).

3.	Comments on the main MS:

A.	Method section: Study area is well written and excellent. You may connect my previous questions (low malaria transmission period in the country to the local contexts).

–	Line 98: Figure 1: Map of the study sites (26): Reference 26 is formatted incorrectly. Please use the standard reference style for websites and ensure it conforms to the journal's guidelines. Alternatively, you may source and cite the map from official national data.

Thank you for flagging the error. We created the maps from an open source, OpenStreetMap for base map. We have corrected the citation according to the guideline of the journal (Line #549-550).

–	Additionally, reference 25 is also incorrect. For example, the "Ethiopian Statistical Agency" should be updated to its current name, the "Ethiopian Statistical Service."

Thank you for the comment. We appreciate you pointing out the potential for an up-to-date citation. However, the document we've referenced is published in 2013 and we've cited it exactly as it appears in the source document to maintain accuracy.

–	Line111-116: Please use this subtitle as “Sampling Design, Sampling technique and Study Population”:

We have accepted the suggestion and made the changes in the revised manuscript. (Line #125).

o	Line 112: Please start with: A community-based cross-sectional survey was conducted between January 30….. Because you collected your data from the community (included individuals from households). People who are reading your work will pay attention. Make sure also you stated in a similar fashion in the abstract section.

We have accepted the suggestion and made clarification in the revised manuscript. (Line # 30 and 131).

o	Sampling technique: Please include whether you used a Systematic random sampling (SRS) in this section. It makes this section complete and readable.

Yes, we have used simple random sampling of the households as it is implemented in STATA software . The sampling was based on the map of the towns. We used ArcGIS (version 10.8) to divide the towns into 100m grids then the centroids of the grids were generated. From the list of coordinates, we used stata to select 83 HH randomly. We have edited the section to clarify the process. (Line #131-138).

o	Line 113: please replace “parasitemia” with “infection”.

Thank you for the correction. The edits are done in the revised manuscript (Line #132).

o	Sample size determination: Not clear. Please detail the methodology used to arrive at 277 households. Furthermore, explain the steps for selecting 912 individuals from those 277 households and three towns.

We accepted the comments and moved the sample size determination to sample design section and clarified the process. (Line #126-130).

o	Line 125-: Please clearly include inclusion and exclusion criteria of your study participants. This will enhance clarity of your study.

The inclusion criteria to participate in the study was being a member of the randomly selected household whose age is more than 6 months and providing consent and/or assent. (Line #139).

–	Line 138 (Section Molecular and Serological analyses), please remove the word “conducted “.

We accepted the suggestion and made the changes in the revised manuscript (Line #160).

–	Line 163 -168 likely address sample size determination, which is a prerequisite planning step and not a part of the statistical analysis conducted on your collected data. You should therefore cut this content from Lines 164–168, create a new subheading titled "Sample size determination," and paste this entire section between line numbers 124 and 125 to improve the flow and organization of your Methods section.

Thank you very much for this great comment. We have accepted the suggestion and made the edits in the revised manuscript (Line #126-130)

–	The Statistical analysis section should start from line 169.

We accepted the suggestion and made the changes in the revised version of the manuscript. (now starts at Line #191)

–	Line 170: Please write out the full term for the abbreviation Finite Mixture Models (FMM) upon its first mention.

We accepted the comment and made the changes in the revised manuscript. (Line #192)

–	Line 172: as suggested above, do the same for MFI.

We accepted the comment and made the changes in the revised manuscript. (Line #194)

–	Line 182-187: please provide reference (citation) for both statistical models you used.

We have added appropriate citations to support both statistical approaches mentioned in Lines #191-195. For the finite mixture model (FMM) with normal distributions and maximum likelihood estimation, we included a reference of McLachlan and Peel (2000), a foundational text on finite mixture models, along with relevant methodological literature supporting its application in serological data classification (i.e. reference 38 and 41). (Line #578-584).

Additionally, we included a citation for the GEE model. Andreas Ziegler (2011) Generalized Estimating Equations (Line #607)

–	Line 202 and 204: Please provide valid references for the software you used. This is acknowledging their works.

We have accepted the comment and added appropriate citations to acknowledge the software tools used in our analysis, R software: R Core Team (2023). *R: A language and environment for statistical computing*. R Foundation for Statistical Computing, Vienna, Austria. Available at: [https://www.R-project.org](https://www.r-project.org/) (Line# 226) (Line# 612)

B.	Results section:

–	Line 216: Kindly replace the subheading "Household and Participant Characteristics" with the more precise phrase: "Household and Sociodemographic Characteristics of the Study Participants.

We accepted the comment and made the changes in the revised manuscript. (Line #239)

–	In this result section, it is a standard practice to present the Sociodemographic Characteristics of the Study Participants before presenting the general Household Characteristics. Please reorder your content accordingly. Specifically, the table currently labeled Table 1 should be renumbered as Table 2, and the table currently labeled Table 2 should become Table 1. So, your paragraph one must Sociodemographic Characteristics of the Study Participants and etc.

We appreciate the reviewer’s attention to presentation standards and the suggestion to reorder Tables 1 and 2. While presenting individual sociodemographic characteristics first is common, our current order was intentionally chosen to align with the data collection hierarchy and analytical framework. The Household Characteristics table (Table 1) provides essential contextual information such as location, wealth, and size that frames the study population before detailing the Sociodemographic Characteristics of individuals (Table 2). Presenting the broader context first clarifies the structural factors influencing participants. Therefore, we respectfully request to retain the current table order and numbering.

–	Line 232: Please correct as: A total of 912 individuals were included in the surveyed households, of whom 51.2% (n=467) were male.

We accepted the comment and made the changes in the revised manuscript. (Line #258)

–	Line 240: rewrite the subheading as Prevalence of malaria infection.

We accepted the comment and made the changes in the revised manuscript. (Line #271)

–	Line 244: 2.3% P. vivax. Please press the pace bar between % and P. vivax as 2.3% P. vivax.

We accepted the comment and made the changes in the revised manuscript. (Line #275)

–	Line 250: Why the total number of individuals included in your Serological testing was 767? Why not 774? Please justify this?

The number of samples tested using RDT and serology varied because we were unable to collect enough blood samples for serological analysis from few child participants (n=2). Additionally, we lost few samples during storage.

–	Line 250: Please include the seroprevalence of malaria as 19.4% (n=767) in your abstract section.

We accepted the comment and made the changes in the revised manuscript. (Line #35-37)

–	Line 260: Please correct the caption for Table 3. The current caption, "Fever and malaria prevalence," is too brief. Please follow standard academic format by including the essential context (e.g., location, time, or participant group). For example: Table 3. Fever and prevalence of malaria in selected towns within Eastern Oromia Regional State, Ethiopia, January to March, 2020.

We accepted the comment and made the changes in the revised manuscript. (Line #291-292)

C.	Discussion Section:

–	It is well written; result interpretations and pertinent justifications are provided.

–	Line 407-412: Studies have documented widespread... The claim of "widespread documentation" is currently supported by only a single reference (68). Please strengthen this statement by adding multiple relevant citations besides number 68. At the same time, please go through this section and strengthen your synthesis and justifications with more references (citations).

Thank you for the feedback. We have now referenced four studies among these one is a review paper in the revised manuscript (see reference 74-78). (Line #677-689)

4.	Important questions

Line 419-422: Your recommendations: “To address these challenges, malaria control programs must adopt more adaptive, data-driven strategies. This includes deploying next-generation tools such as dual-active ingredient nets, rotating insecticides for IRS, and enhancing community education to promote consistent ITN use.”

A.	Is this the only recommendation from your study for the National malaria control programme of Ethiopia?

We have included recommendations such as targeted larval source management and environmental interventions based on the risk factors identified (Line # 427-429). Additionally, we showed that integrating serological and spatial clustering analysis within the surveillance can identify hotspots which will inform targeting of interventions (line #478-480). In the revised manuscript, we have rephrased the statement to clarify the recommendations.

B.	Don’t you think what you are recommending are already not in place?

These recommendations are already in the national malaria strategy. However, the reach to urban areas is limited. This might be due to resource constraint. .

C.	Your findings show that RDT-based prevalence of malaria is only 0.78% (n=6) while that qPCR is 4% (n=31).

1)	The data show a significant discrepancy: RDT-based prevalence is very low at 0.78% (n=6), yet qPCR-based prevalence is five times higher at 4% (n=31). Given these findings, what does this mean for the operational goals of the current National Malaria Elimination Strategic Plan (2024/25-2026/27)? Specifically, how might this "hidden reservoir" of infection (detected only by qPCR) affect elimination targets and surveillance strategies relying primarily on RDTs?

We thank the reviewer for requesting clarification on implication of the results. The discrepancy of RDT prevalence 0.78% (n=6) vs qPCR prevalence 4% (n=31) indicates a substantial sub patent (low-density), likely asymptomatic reservoir that RDTs cannot detect (i.e below the 100 parasite per microliter threshold). That “hidden reservoir” has direct consequences for elimination ambitions: it can sustain residual transmission, mask hotspots, and cause over-optimistic assessments of elimination programme progress if screening of cases at elimination districts is guided by RDT results. Cognizant of this issue the Federal Ministry of Health has assessed targeted mass drug administration (tMDA) versus RDT test and treat strategies and included tMDA in elimination settings. However, implementation is still in the pilot phases. We have included this in the discussion section of the revised manuscript (Line# 379-384)

2)	What is the likely scientific and/or biological explanation for the five-fold difference in prevalence between the two diagnostic tools?

The discrepancy is explained by the differences in diagnostic sensitivity. Rapid Diagnostic Test (RDTs) detect parasite antigens such as histidine-rich protein 2 (HRP2) or lactate dehydrogenase (pLDH) in peripheral blood. Its detection limit is ≥100–200 parasites/µL of blood (some sensitive types ~50 parasites/µL). Infections with very low parasite densities fall below this threshold and are therefore missed by RDTs. However, quantitative polymerase chain reaction (qPCR) amplifies parasite DNA and detects even a few copies of parasite nucleic acid. The detection limit can go as few as 0.02–1 parasite/µL of blood. While it is highly sensitive, it is also very expensive and requires a sophisticated laboratory setup, making it unsuitable for routine case detection. We have included this in the discussion section of the revised manuscript (Line# 379-384)

3)	Based on the above analysis, what specific recommendations do you propose regarding diagnostic policy and surveillance strategies in the context of the National Malaria Elimination Plan? Ensure these points are explicitly integrated into the Discussion and Recommendation sections of your manuscript.

qPCR and serological tests are recommended primarily for surveillance rather than for diagnosing individual infections, as they are costly and require sophisticated laboratory infrastructure. Additionally, serology cannot be used for routine diagnostic purposes. Based on our findings, we recommend incorporating serological testing into surveillance activities since it offers a feasible approach for detecting cumulative exposure within populations. We suggest the use of short-lived antibody markers, which better reflect recent transmission. By contrast, qPCR is more suitable for identifying current infections rather than past exposure. We have discussed the low transmission setting in our study area and the heterogeneity and made a recommendation to include serology within the surveillance. We have made clarification on the specific recommendation of short-lived marker on the revised manuscript (Line # 478)

5.	General comments:

Referencing and Citation Style: Please conduct a thorough, final review of the entire References section and in-text citations to ensure absolute consistency. All necessary corrections should be implemented as detailed in the previous specific comments regarding style (e.g., journal name abbreviations, punctuation, and author formatting.

Manuscript Page Numbering: For ease of review and standard formatting, please ensure that all page numbers are consistently positioned at the bottom-center of every page.

We have accepted the comment and made changes on the revised manuscript.

**Response to Reviewer #2**

General:

I have carefully read the document it is well organized and written. The authors investigated urban malaria using Microscopy, PCR and serology methods and reported heterogeneity and recommended tailored intervention approaches.

While the work is good its main drawback is the sampling sites, the three towns selected are relatively in close spatial proximity with similar eco-epidemiology characteristics and all located in the single corridor that connect Djibouti and Addis. They are also connected and supported by single river. Not sure if this is purposive, otherwise, this limits the generalizability of the study.

We agree that the study sites are spatially closer, on the route that connect Djibouti and Addis and utilize the Awash river (The Awash River basin, spanning 23 administrative zones, covers 10% of Ethiopia's area) for irrigation. However, these towns have different transmission category based on stratification of the Ministry: Metehara is classified as high transmission with API 52.8 (API≥50/1000 population), and while Awash Sebat Kilo, API 41 is classified as moderate (API≥10<50) and Adama with API 2.5 is classified as very low (API>5 <10) transmission strata in 2019. Additionally, the communities have differences in the way of living Eg. In Awash Sebat Killo, the community is Agro- pastoral, whereas in Adama and Metehara the residents are more of urbanized and the communities are dependent on service and industry. The selection is purposively done and effort was made to include different transmission status and operationally feasible to conduct the surveys. We have included this as a limitation in the revised manuscript (Line #471-472)

Comments: Considering the below comments may improve the MS

- Authors indicate Ethiopia is the 4th largest contributor globally and later in line 67 they state elimination goal by 2030, is it feasible and relevant mentioning it in the MS? And would this elimination plan for 2030 a targeted elimination in low transmission settings?

We included the most recent national data to inform readers about the current malaria situation in Ethiopia. Although progress has slowed in recent years, the Government of Ethiopia remains committed to achieving malaria elimination in designated low-transmission districts. Our study was conducted in 2020, a period when the country reported relatively lower malaria burden. The primary aim was to assess risk factors in urban areas classified by WHO as low-transmission settings. By identifying context-specific risks, our findings help guide the tailoring of interventions to further reduce transmission in these areas and support Ethiopia’s broader elimination goals.

- Authors may want to indicate intervention interruption among the factors for malaria dynamics ex. Current outbreak/resurgence may have been influenced by intervention interruption.

As mentioned above our focus was to just inform the current situation in the country as we are publishing this result now. We have accepted the suggestion of including the possible reasons for resurgence and made changes in the revised version. (Line #65-66)

- Line 50-52 sounds to have some redundancy with first paragraph

We presented Ethiopia’s 2019 malaria status because it was a period when we planned to conduct the study and of relatively low transmission. This aligns with the objective of our study—to identify risk factors in low-transmission settings and provide programmatic insights for further reducing malaria burden. However, beginning in 2020, the situation changed significantly due to multiple challenges, including conflict and emerging biological threats such as HRP2/3 deletions and the spread of *Anopheles stephensi.* To ensure coherence with the subsequent paragraph that reports the rise to approximately five million cases, we retained this contextual statement to provide a clear and logical transition between the two sections.

- The data collection period Jan to March is out of transmission season, unless there is a reason this may bias the burden estimation.

Yes, we understand the survey was done in dry season. In low transmission settings conducting malaria prevalence in dry season provides a baseline estimate of residual transmission or persistent parasite carriage within the population. These infections represent long-term carriers who maintain the parasite reservoir until the next transmission season which is critical for elimination programs. We have indicated the season in the revised manuscript (Line#131)

- The study uses pfhrp2 RDT but does not consider the effect of hrp discussion that may affect diagnosis accuracy

The initial detection of pfhrp2/3 deletion in the northwestern region was reported in 2020 (Fe[leke et al 2020](https://www.nature.com/articles/s41564-021-00962-4)), with subsequent surveys confirming its expansion ([Migbaru et al., 2024](https://papers.ssrn.com/sol3/papers.cfm?abstract_id=4938046)). However, our study was conducted in 2020, when pfhrp2/3-based RDTs were still the primary diagnostic tool in use. To ensure accurate infection and exposure assessment, we employed Polymerase Chain Reaction (PCR) and Serology. These methods, which detect parasite nucleic acids and exposure markers, respectively, are the diagnostic choice for assessing infection and risk in low-transmission settings. Therefore, the potential impact of pfhrp2/3 deletions on our findings is negligible.

- Consider uniformity in methods either describe details or reference ex line 134 either write the extraction procedure in short or make it full. Similar comment may hold for PCR and serology assays

We accepted the suggestion and made changes in the revised version of the manuscript. (Line #157 and 177)

- Line 136-139 consider revising language

We accepted the suggestion and made changes in the revised version. (Line #157-160)

- Line 156 antigen coupling, this is relevant either describe it or reference it

Accepted the suggestion and made changes in the revised version. (Line # 177)

- Line 172 define MFI

Accepted the suggestion and made changes in the revised version. (Line #194)

- Line 207 what does approved by AHRI means consider language revision

Thanks for flagging it. We corrected it as “ethical clearance for the study was also obtained from Ethics review Board at Armauer Hansen Research Institute”. See the changes in the revised version (Line #230)

- Line 223 television add % for consistency

We have accepted the comment and made changes on the revised manuscript. (Line# 247)

- Line 225/26 consider the use of only, better reporting as is

We have accepted the comment and made changes on the revised manuscript. (Line# 251)

- Table 1 the word improved is confusing ex. What is improved roof …is it corrugated iron roof or what?

We defined improved in the variables as below

- Improved water source: dug well or piped water, or bottled water
- Improved sanitation: flush or pour toilet; or Pit latrine
- Improved cooking fuel: use of electricity and/or Kerosin
- Improved floor material: made of cement, ceramic or polished wood
- Improved roof material: made from metal, or wood or cement

The definitions are included in supporting information document

- Line 233 consider median age and IQR

We have accepted the comment and made changes on the revised manuscript. (Line# 259)

- Line 304/6 would the relation of infection vs IRS/ITN may be affected by previous exposure or infection history

Yes, past exposure will impact current infection through immunity and lead to more asymptomatic infection. On the other hand, if IRS coverage and ITN utilization is high then it will impact infection through the reduction of vector-human contact ([Lengeler](https://www.cochranelibrary.com/cdsr/doi/10.1002/14651858.CD000363.pub2/full) *[et al.](https://www.cochranelibrary.com/cdsr/doi/10.1002/14651858.CD000363.pub2/full)*[2004](https://www.cochranelibrary.com/cdsr/doi/10.1002/14651858.CD000363.pub2/full))

- In high-exposure (stable transmission) areas, ITNs reduced the incidence of uncomplicated malarial episodes by 50% compared to no nets. Additionally, ITNs had an impact on severe malaria, parasite prevalence, high parasitaemia, splenomegaly, and their use improved the average haemoglobin level in children by 1.7% packed cell volume.
- In low-exposure (unstable transmission) areas, ITNs reduced the incidence of uncomplicated malarial episodes by 62% for compared to no nets.

- Line 341 discussion consider referencing this document: 10.1186/s12936-019-2874-z.

We accepted the suggestion and included as a reference in the discussion (Line #387-388 and 395)

- Line 377 is 1712 that high to affect malaria transmission?

Yes, malaria transmission decreases as altitude increases. A national seroprevalence study in Ethiopia showed that *P. falciparum* seropositivity was inversely associated with elevation. The highest seropositivity occurred at elevations below 1,500 meters, with a marked peak at altitudes under 1,000 meters (Assefa et al 2019).In our study there is marked elevation difference between our sites Adama (1712m) compared Awash Sebat Kilo (916m) and Metehara (947m).

Additionally, a study by Bødke et al 2003 from Tanzania showed:

- Sporozoite rates in *Anopheles gambiae s.l.* decreased by about 4-fold from 300 m to 800 m (during the peak season). Rates were impossible to measure in the two highest villages (1,400 m and 1,700 m) due to extremely low vector densities.
- Total transmission intensity was reduced by approximately 1000-fold between the holoendemic lowland (300m) and the hypoendemic highland plateau (1700m). The estimated Basic Reproductive Rate (R0) dropped from 1,841 at 300 m to below 1 at 1,700 m, indicating unsustainable transmission at the highest elevation.
- Man biting rate dropped by 50% for every 126-meter increase in altitude.

- Line 395 what about increase on age as a risk factor for infection?

Our findings indicate that adults had a higher risk of exposure, as demonstrated by the serological markers, particularly the short-lived antibodies. However, because the number of infections detected in our study was very low, we were unable to perform a meaningful regression analysis to assess age as a risk factor for current infection using PCR or RDT.

- Line 405 resistance to deltamethrin may reduce efficacy but cannot increase infection, please elaborate

Resistance to deltamethrin can reduce the effectiveness of vector control tools such as insecticide-treated nets and indoor residual spraying; however, resistance itself does not directly increase malaria infection. Instead, reduced susceptibility in mosquitoes may diminish the protective effect of these interventions, potentially allowing more mosquito–human contact. This indirect pathway can contribute to higher transmission risk.

- Try to increase picture resolution for all and put pictures to scale

We have accepted the suggestion and increased the resolution of the figure. However, we would like to retain the scale used in Supplementary Fig. 1. A different scale was chosen for Adama because the data would not be visible if we applied the same 0–100 scale used for Metehara and Awash Sebat Kilo. Footnote describing the different scale is added in the supporting information document.

- Consider typo totmal/Total supplement figure 1

We accepted the suggestion and made changes in the Figure legend of supplement figure 1

**Response to Reviewer #3**

1) This is an interesting paper exploring the use of serologic markers for fine scale epidemiological mapping of malaria transmission heterogeneity in urban settings. It also aimed to identify risk factors.

Perhaps the title should state “Heterogeneity of malaria transmission….” adding the key word “transmission”. It is also possible to suggest a modified title since heterogeneity is natural and expected. Just an example “A sero-prevalence and risk factor study of urban malaria in Ethiopia”.

We appreciate the reviewer’s suggestion regarding the title. While heterogeneity is indeed an expected characteristic of malaria transmission, the degree of heterogeneity and its highly localized pattern within this urban setting represent a central, policy-relevant finding of our study. We have therefore partially accepted the suggestion and revised the title (Line #1) to enhance clarity and flow while preserving the core message. Specifically, we chose to retain the phrase “heterogeneity of transmission” because it concisely captures our most important result and directly informs targeted control strategies.

2) While exploring potential risk factors, the authors could have taken account of physico-environmental (altitude, mean monthly temperature, etc.), population size/density, and vector parameters and species in the analysis of the data. It seems all the three towns have similar agro-climatic setting and are close to Awash River and the associated irrigation agriculture schemes. Just wondering if the choice of study sites was ideal for the intended purpose. High transmission towns or a high transmission season could have been opted.

We agree that environmental and entomological factors should be examined in these settings. Next steps include entomological and ecological assessment of risk in these same setting.

We also agree that the study sites are spatially closer, on the route that connect Djibouti and Addis and utilize the Awash river (The Awash River basin, spanning 23 administrative zones, covers 10% of Ethiopia's area) for irrigation. However, these towns have different transmission category based on Federal Ministry of Health (FMOH) stratification, Metehara is classified as high transmission with API 52.8 (API≥50/1000 population), and while Awash Sebat Kilo, API 41 is classified as moderate (API≥10<50) and Adama with API 2.5 is classified as very low (API>5 <10) transmission strata in 2019. Additionally, the communities have differences in their way of living Eg. In Awash Sebat Killo, the community is Agro- pastoral, where as in Adama and Metehara is more urbanized and the communities are dependent on service and industry. The selection is purposively and tried to include different transmission status and operationally feasible to conduct the surveys. (Line #119-124)

Additionally, conducting malaria prevalence in dry season and in low transmission settings, provides a baseline estimate of residual transmission or persistent parasite carriage within the population. These infections represent long-term carriers who maintain the parasite reservoir until the next transmission season which is critical for elimination programs. We have included this in the discussion section of the revised manuscript (Line# 379-384)

3) The authors have rightly pointed out the key limitation of the study, i.e., sample size. First and foremost, the geographic variations (location) are not accounted for, and the assumption about prevalence (10% PCR positivity) and household size of 3 persons per HH was not well thought of. It is also unclear how household numbers were partitioned between the three towns, with higher number of households sampled in Awash where the total population was the least and ultimately a higher prevalence was found in this town. These estimates would probably be valid for the high transmission season in these localities. In addition, there was a high dropout rate (81 households not sampled). It seems the risk of bias could be high. Given this, the authors could have softened the tone of the conclusions. It is impossible to make hard conclusions and at the same time highlighting significant limitations. In line 342, they mention that the study has given ‘new insight about malaria transmission dynamics”. Furthermore, in lines 354/355, they also mention the serological data has allowed a deeper understanding of exposure. I think that study has given an insight that might help future large-scale studies, but it is far from the provision of a deep understanding of exposure.

We thank the reviewer for their careful assessment and valuable feedback regarding the limitations of our study, particularly concerning sample size, sampling strategy, and the interpretation of our findings. We acknowledge that these concerns are critical and have revised the manuscript accordingly to address them.

1. Assumptions on Prevalence and Household Size

Prevalence (10%): this estimate was based on data from Adama which one of our site([Fitsum et al](https://malariajournal.biomedcentral.com/articles/10.1186/s12936-017-1749-4)) available local urban estimate. In addition, household size (3 persons/HH) is derived from the official Ethiopian Health and Demographic Survey (DHS) for urban households, conducted by the Central Statistics Agency ([Central Statistics Agency](chrome-extension://efaidnbmnnnibpcajpcglclefindmkaj/https:/www.unicef.org/ethiopia/media/456/file/edhs%202016.pdf)). The sources were referenced in the manuscript.

2. Household Partitioning and Sample Distribution

The reviewer questions the partitioning of households between the three towns, noting the seemingly high prevalence found in Awash. Our initial calculation used the population proportion formula to achieve a total target sample size of 249 individuals, per town (249 / 3 (family size) = 83 households per town). We tried to oversample (110HH) in Afar as we have coordinates were not inhabited (that was randomly selected) due to practical challenge encountered in Afar's dispersed settlement patterns. We learned while generating coordinates (samples) using town-base maps alone will lead to the selection of uninhabited areas thus we think overlaying a polygon of google map of coordinates to generate spatial map of all inhabited households will be important in the future studies. The higher prevalence in Awash, despite its population size, is an empirical finding that we attribute to the unique transmission dynamics of that specific locale, which strengthens the need for further, larger-scale investigation rather than negating the data.

3. Dropout Rate and Risk of Bias

We fully agree with the reviewer that the high dropout rate (81 households not sampled) poses a significant limitation and increases the risk of bias. We have expanded our discussion of this limitation, explicitly mentioning the geographical sampling difficulties (particularly the need to sample households outside the town's administrative boundary) introduce potential selection bias. The high dropout rate is now a central feature of the revised Limitations paragraph, acknowledging that our findings may not be perfectly generalizable.

4. Softening the Tone of Conclusions

We accept the suggestion to soften the tone of the conclusions to align the discussion with the acknowledged methodological limitations.

We have revised the text in the discussion sections to use more cautious and suggestive language. “This study provides new insights into malaria transmission dynamics in three towns in Ethiopia” “new” is deleted (Line #373)

The mention of serological data allowing a "deeper understanding of exposure" has been softened to "The integration of serological data provide an understanding of exposure and spatial heterogeneity.” (Line #391). These revisions ensure the discussion appropriately reflect the modest scale and limitations of the investigation.

4) Some discussion points are speculative and unsharpened. For instance, the issue of data quality (lines 362-363) implicates poor quality in Afar and better quality in Oromia. While this assessment could be true, it barely qualifies as a valid statement when not supported by evidence. The authors are seeking potential explanations for observed events, but for simplest explanations to complex phenomenon. In line 366, they refer to the success of control efforts in Metehara to explain recent declines (among young individuals) in malaria prevalence. This also requires a proper assessment of control efforts and its impact, but also by extending the assessment of seroconversion rates over different time points especially as we know malaria transmission varies seasonally and geographically. The success of a control program can be better evidenced preferably by measuring and finding a decline in sero-conversion rates over repeated time points, and statistically by determining the likelihood ratio. Similarly in lines 413-417, the discussion of reverse causation in explaining the higher odds of exposure to malaria in protected individuals/communities is not successful. It might just help posing questions whether such situations are explanatory.

Quality issues in routine surveillance data

For Awash Sebat Town which is in Afar, it is classified as moderate using surveillance data that we collected from the Ministry of Health. However, the serological data showed high transmission. One possible explanation can be the issue of data quality as it impacts malaria stratification based on routine surveillance data. The issue of poor quality in Afar and better quality in Oromia is documented in the reference we cited. (Reference: *Ethiopian Public Health Institute FMoHaWHo. Ethiopia Health Data Quality Review: System Assessment and Data Verification for Selected Indicator. Addis Ababa Ethiopia: Ethiopian Public Health Institute 2016*)

Use of serology to understand the impact of interventions

Serological markers are valuable for assessing the impact of interventions. The Seroconversion Rate (SCR), derived from age-stratified serological data, estimates the Force of Infection and serves as a strong proxy for malaria transmission. In our study, we calculate age-stratified SCR using maximum likelihood estimation. The SCR’s value lies in its ability to reconstruct historical exposure from a single cross-sectional survey using the Seroconversion Rate Change-Point Model. This model identifies the time at which the SCR most likely shifted, often corresponding to major public health interventions. Thus do not need multiple data points over multiple years.

Reverse causation

Reverse causation arises when an outcome influences exposure, rather than the exposure influencing the outcome. In our case owning ITN causes higher malaria exposure risk which is counter intuitive as we are expecting the risk to be lower in households with preventive intervention. We have provided reference to similar findings from Ethiopian settings where universal coverage was not possible in one campaign and ITNs must be prioritized to higher risk areas.

5) In lines 349-351, the authors also discuss the higher prevalence of P. vivax than P. falciparum as a typical phenomenon in low transmission seasons. This is probably partially true. But, since most of the tested individuals were asymptomatic, there is also high chance that asymptomatic parasitemia is contributed largely by P. vivax even in high transmission seasons or localities.

In Ethiopia the prevalence of asymptomatic malaria is largely depends on the intensity of the transmission. In higher transmission setting *Plasmodium falciparum* is the dominant species [Hailemeskel et al. 2021](https://malariajournal.biomedcentral.com/articles/10.1186/s12936-021-03587-4). Where as in areas where *Plasmodium falciparum* is controlled i.e low transmission setting *P. vivax* will be the dominant species due to its unique biological traits such as the ability to form hypnozoites, early gamete formation prior to showing symptoms etc.. see the study by [Habtamu et al](https://doi.org/10.1186/s40794-022-00185-3). and [Price et al 2020](https://pubmed.ncbi.nlm.nih.gov/32407682/) (Line #389-392)

6) There were 91 individuals (10%) who had fever in the two weeks period prior to sampling. It would have helped what proportion of these were RDT/Serology/PCR positive for malaria cf. total population. A similar analysis of data for individuals who had slept under the net cf. those who have not, might also give an insight of exposure patterns. Such data seems to be available in figure 3, but the title and the legends of figure 3 do not indicate what tests were used to determine exposure and to ultimately calculate OR. Such data might help to resolve the discussion of paradoxes, e.g., the higher odds of exposure in protected individuals and provide opportunity for alternative explanations. It is also possible that sero-positivity may be associated with protection from repeated exposure (due to acquired immunity), and the increased odds may be obscure rather than paradoxical.

We agree that examining the proportion of individuals with reported fever who were RDT- or PCR-positive for malaria, as well as the proportion who slept under an ITN, could offer useful insight into exposure patterns. However, the number of RDT- and PCR-positive cases in our dataset is too small to support meaningful analysis. For this reason, we selected serology as the basis for our risk factor analysis.

In Figure 3, we used short-term serological markers for the analysis, as the number of PCR- and RDT-positive cases was very low, which was expected given the dry season and the low-transmission setting. While the association between ITN use and seropositivity could be explained by historical exposure when using long-lived markers, our analysis was based on short-lived markers that reflect recent exposure within the past 9–12 months. Therefore, the observed associations are likely to represent recent rather than historical exposure.

Minor comments

1) In line 53, the number of malaria cases is given in 6-digit number and then the word “million” is included. In addition, the authors sequence the year and number of cases (i.e., “In 2023, 5,677,912”). This could be tidied by restructuring the sentence and alternating the sequence of numbers. For example, it can be stated as “In the Ethiopian HMIS, there were reports of 5,677,912 malaria cases in 2023, accounting for ……….,”. Similarly, in line 222-223, the statement “Most households owned mobile phones 95% (n=186) and a television (n=164)” which sequences words and percentage. I suggest the sentence to read as “Most households owned mobile phones (n=186, 94.9%) and television (n=164, 83.7%). This is a common problem in the many manuscripts that we have reviewed previously. Another example is in lines 226-227, whereby sequencing is noted. The statement “Most households had improved roofing 87% (n=171) and flooring 76% (n=149) materials, 31% (n=61). The problem is the sequence “flooring 76%” and “roofing 87%”, and the 31% is supposed to be for eaves not included in the statement. It is better written as “Most households had improved roofing (n=171, 87%) and flooring (n=149, 76%) materials and eaves (n=61, 31.1%).

Thank you for catching the typo and statement restructuring suggestions. We have made the corrections in the revised manuscript (Line #68-69) and (Line # 243-255).

2) In lines 267-269, seroconversion rates of 0.15, 0.035, 0.002, 0.063 are mentioned. Are these percentages or proportions? They appear to be “proportions” without the % symbol.

No, they are not percentages. The figures are expressed as rates. The rate at which individuals convert from seronegative to seropositive status per year.
