# Supplementary material for: Heterogeneity of malaria transmission in urban settings in Ethiopia: A seroprevalence and risk factor analysis
Source: PLoS One. 2026 Feb 5;21(2):e0328118. doi: 10.1371/journal.pone.0328118 (PMC12875449; doi:10.1371/journal.pone.0328118)
Supplement: Attachment — Submitted filename: My feedback for Manuscript_PONE-D-25-32925R1.docx [file pone.0328118.s008.docx]

**MY FEEDBACK FOR PLOS ONE**

**MS reference number: PONE-D-25-32925R1**

**Corresponding Author: Hiwot Teka Namaga**, Addis Ababa University, Addis Ababa, ETHIOPIA

**General Reflection on the Work**

**I reiterate my previous feedback’s introduction** as follows: -

the authors have produced a highly exhaustive, timely, and impactful study on the emerging challenges of urban malaria in Ethiopia. This work has the potential to significantly contribute to the country’s ongoing malaria elimination efforts.

The authors have also addressed my queries ***point-by-point***, providing sound scientific and biological justifications. I appreciate the thoroughness of their literature review and the resulting improvements to the manuscript. Given the depth of my previous feedback, I find the authors' current revisions to be more than satisfactory.

**Minor Suggestion**

My only remaining suggestion concerns the notation for the **Adjusted Odds Ratio (AOR).** Currently, the authors use "OR," which typically denotes a Crude Odds Ratio (COR). As inferences should be based on the adjusted model, I recommend the authors consistently use the abbreviation AOR to clearly indicate that the measure accounts for potential confounders.
